# Physical activity and mental well-being under COVID-19 lockdown: a cross-sectional multination study

**DOI:** 10.1186/s12889-021-10931-5

**Published:** 2021-05-27

**Authors:** Costas I. Karageorghis, Jonathan M. Bird, Jasmin C. Hutchinson, Mark Hamer, Yvonne N. Delevoye-Turrell, Ségolène M. R. Guérin, Elizabeth M. Mullin, Kathleen T. Mellano, Renée L. Parsons-Smith, Victoria R. Terry, Peter C. Terry

**Affiliations:** 1 Department of Life Sciences, Brunel University London, Middlesex, UB8 3PH UK; 2 Department of Science, Innovation, Technology, and Entrepreneurship, University of Exeter, Exeter, UK; 3 Department of Exercise Science and Athletic Training, Springfield College, Springfield, MA, USA; 4 Institute of Sport, Exercise & Health, Research Department of Targeted Intervention, University College London, London, UK; 5 Univ. Lille, CNRS, UMR 9193 - SCALab - Sciences Cognitives et Sciences Affectives, F-59000 Lille, France; 6 School of Psychology and Counselling, University of Southern Queensland, Toowoomba, Australia; 7 School of Health and Behavioural Sciences, University of the Sunshine Coast, Sunshine Coast, Australia; 8 School of Nursing and Midwifery, University of Southern Queensland, Toowoomba, Australia; 9 Division of Research and Innovation, University of Southern Queensland, Toowoomba, Australia

**Keywords:** Coronavirus, Exercise, International, Mental well-being, Pandemic

## Abstract

**Background:**

COVID-19 lockdowns have reduced opportunities for physical activity (PA) and encouraged more sedentary lifestyles. A concomitant of sedentariness is compromised mental health. We investigated the effects of COVID-19 lockdown on PA, sedentary behavior, and mental health across four Western nations (USA, UK, France, and Australia).

**Methods:**

An online survey was administered in the second quarter of 2020 (*N* = 2541). We measured planned and unplanned dimensions of PA using the Brunel Lifestyle Physical Activity Questionnaire and mental health using the 12-item General Health Questionnaire. Steps per day were recorded only from participants who used an electronic device for this purpose, and sedentary behavior was reported in hours per day (sitting and screen time).

**Results:**

In the USA and Australia samples, there was a significant decline in planned PA from pre- to during lockdown. Among young adults, Australians exhibited the lowest planned PA scores, while in middle-aged groups, the UK recorded the highest. Young adults exhibited the largest reduction in unplanned PA. Across nations, there was a reduction of ~ 2000 steps per day. Large increases in sedentary behavior emerged during lockdown, which were most acute in young adults. Lockdown was associated with a decline in mental health that was more pronounced in women.

**Conclusions:**

The findings illustrate the deleterious effects of lockdown on PA, sedentary behavior, and mental health across four Western nations. Australian young and lower middle-aged adults appeared to fare particularly badly in terms of planned PA. The reduction in steps per day is equivalent to the non-expenditure of ~ 100 kcal. Declines in mental health show how harmful lockdowns can be for women in particular.

**Supplementary Information:**

The online version contains supplementary material available at 10.1186/s12889-021-10931-5.

## Background

COVID-19 is a highly contagious disease related to severe acute respiratory syndrome coronavirus 2 (SARS-CoV-2). Once the outbreak of the disease was categorized as a global health pandemic in March, 2020 [1], extensive social distancing and isolation policies (eg, lockdowns) were employed by governments to reduce the strain on health services. Lockdowns have severely limited opportunities for physical activity (PA) [2]. The health benefits of PA include reduced risk of cardiovascular disease, hypertension, diabetes, and some cancers [3]. The closure of businesses, schools, and community facilities (eg, public parks) has encouraged sedentary behavior during the pandemic [4]. High levels of sedentary behavior, typically assessed via daily sitting and screen time, are associated with greater risk for all-cause mortality, cardiovascular disease, type 2 diabetes, and some cancers [5, 6].

It has been estimated that 970 million people worldwide suffer from mental health problems, such as depressive and anxiety disorders [7]. Accordingly, the stressors associated with COVID-19 (eg, inability to see loved ones, job uncertainty) are likely to augment pre-existing psychological distress in modern society [8]. This is exacerbated by limited opportunities for PA during lockdown, given that such behavior has a positive impact on mental health [9].

Early findings indicated that lockdowns led to a decrease in PA coupled with an increase in sedentary behavior [10, 11], albeit that researchers rarely take direct or objective measures of PA behavior (eg, daily step counts) [12]. In the USA, Meyer et al. conducted a cross-sectional study in which they reported a 32% decrease in PA during the COVID-19 pandemic among adults who had been physically active [13]. Moreover, the researchers detailed that the largest increases in sedentary behavior were associated with those who had been compelled to self-isolate.

Recent findings also illustrate detriments in mental health during the COVID-19 pandemic [14, 15]. For example, Banna et al. administered an online survey to adults in Bangladesh and found that the prevalence of anxiety symptoms and depressive symptoms were 33.7% and 57.9%, respectively [16]. Furthermore, 59.7% of participants reported mild-to-extremely severe levels of stress. Women reported higher anxiety, depression, and stress when compared to men [16]. Similar findings were reported in China and the UK [17, 18].

It is plausible that changes in PA, sedentary behavior, and mental health are not evenly distributed within populations and across nations [8]. Groups of interest include women [18] as well as younger (18–29 years) and older (≥ 60 years) adults [19]. The policies of national governments varied considerably during the COVID-19 pandemic, resulting in disproportionate effects on subgroups of populations within individual nation states (eg, gig economy workers, people of color, and health professionals) [19–21]. Nonetheless, there is limited research that incorporates data spanning multiple nations [22].

The primary aim of the present study was to examine the effects of initial COVID-19 lockdowns across four Western nations with a focus on PA levels, sedentary behavior, and mental health. A secondary aim was to examine age as a moderator but in the case of mental health, we examined sex as a moderator [18, 20]. In each of the analyses, nation membership was included for exploratory purposes.

We hypothesized that there would be reductions in PA dimensions and steps per day, from pre- to during lockdown, and that age would not moderate this trend (*H*_1_). For sedentary behavior, we hypothesized increases from pre- to during lockdown and that age would not moderate this trend (*H*_2_). For mental health, we hypothesized a decline from pre- to during lockdown, with a greater decline among women (ie, a significant lockdown × sex interaction; *H*_3_). The knowledge derived from the present investigation might enable public health practitioners to develop interventions targeted toward the promotion of PA and mental health, coupled with a reduction in sedentary behavior, all of which have been identified as public health priorities [19, 23, 24].

## Methods

### Nations

Four Western nations (USA, UK, France, and Australia) were chosen for the present investigation given the different government policies they employed during the initial COVID-19 lockdown (eg, school closures, access to exercise facilities). State and regional “stay at home” orders were issued in the USA between March 21 and April 7, 2020. The UK and France entered national lockdowns on March 23 and March 16, 2020, respectively. Interstate border closures in Australia began on March 19, 2020.

### Participants

We used a cross-sectional design, with recruitment facilitated by email and social media posts. Volunteer participants were eligible if they were aged ≥18 years; resided in the USA, UK, France, or Australia; and spoke the main language of their country of residence (ie, English or French). An a priori calculation for sample size was not conducted as we (a) had a limited timeframe in which to collect data during the first wave of international lockdowns (ie, we adopted a resource constraints approach [25]), and (b) had no indication of the effect of lockdowns on the parameters of interest in the context of the ‘natural experiment’ created by the global pandemic [26].

A convenience sample of 2541 adults completed the survey (*n*_USA_ = 1029, *M*_age_ = 40.7 years, 786 women, 237 men, five who selected “other”, and one who preferred not to say; *n*_UK_ = 392, *M*_age_ = 51.2 years, 314 women, 77 men, and one who preferred not to say; *n*_France_ = 734, *M*_age_ = 37.7 years, 558 women and 176 men; *n*_Australia_ = 386, *M*_age_ = 42.5 years, 280 women, 104 men and two who preferred not to say; see Additional file 1: Table S1). Participants were administered an information sheet and asked to provide informed consent. The study protocol was approved by the College of Health, Medicine and Life Sciences Research Ethics Committee, Brunel University London (23175-LR-May/2020-25477-1), and data collection in each nation was approved by a local ethics committee. This article follows the STROBE guidelines for the reporting of observational studies [27].

### Measures

A range of demographic data was requested from participants in the first part of the survey. Such data included sex, age, setting (ie, rural vs. urban), and occupational status. We also requested anthropometric data (height and weight), from which we calculated body mass index (BMI). Moreover, we requested health-related data (health conditions and disabilities) as well as COVID-19 symptoms, diagnosis, and recovery details (see Additional file 1: Table S1).

Planned and unplanned dimensions of PA were assessed using the Brunel Lifestyle Physical Activity Questionnaire (BLPAQ) [28], which has nine items attached to 5-point continuous-closed numerical scales (eg, 1 = *Not at all,* 5 = *Highly*). The authors of the BLPAQ defined planned PA as, “… any activity that is scheduled into your daily routine, which may enhance your health, fitness, or well-being” (eg, brisk walking, cycling) [28] (p2). Unplanned PA was defined as any form of PA “excluding pre-planned physical activity” (eg, heavy housework, playing with children) [28] (p3). Sample BLPAQ items are, “In general, what is the duration of each session of pre-planned PA that you engage in?” (planned PA subscale) and “In general, how physically demanding are your job or your day-to-day activities?” (unplanned PA subscale).

Factor scores for planned and unplanned dimensions of PA are calculated by adding scores from items 1–6 (planned) and 7–9 (unplanned), then dividing them by six and three, respectively. Factor scores ranged from 1.00–5.00, with higher scores indicating higher engagement in PA. The BLPAQ has acceptable test–retest reliability and is a criterion- and cross-validated measure of physical activity [29, 30]. Cronbach’s alpha coefficients for planned PA was 0.92 (pre-lockdown) and 0.93 (during lockdown) in the present sample. Alpha estimates were lower for the unplanned PA scale (pre-lockdown = 0.52, during lockdown = 0.64), as is often the case with scales that contain a small number of items [31]. Participants specified their average step-count per day if they used an electronic device to monitor such activity.

Sedentary behavior was measured by asking each participant to provide estimates of their daily sitting time and time spent viewing a screen (ie, two items ranging from 0 to 24 h). A sample item is “Please estimate how many hours per day you typically spend sitting during the COVID-19 lockdown” (sitting item).

Mental health was assessed using the 12-item General Health Questionnaire (GHQ-12) [32], which has 12 items attached to 4-point bipolar scales (eg, 0 = *Better than usual,* 3 = *Much less than usual*). The items concern a variety of psychological constructs, such as anxiety, depression, and social dysfunction. A sample item is, “Have you recently been feeling unhappy and depressed?” A factor score is calculated by adding the item scores. Therefore, possible values span 0–36, with higher scores indicating poorer mental health. The GHQ-12 has demonstrated both convergent validity and internal consistency [33]. Cronbach’s alpha coefficients for mental health were 0.87 (pre-lockdown) and 0.91 (during lockdown) in the present sample.

### Procedure

The ~ 20-min survey was administered via web-based software (Qualtrics; Provo, UT, USA, and LimeSurvey; Hamburg, Germany) and participants were not offered any incentive. We measured PA levels, sedentary behavior, and mental health pre- and during COVID-19 lockdowns in the USA, UK, France, and Australia. A retrospective frame was adopted for pre-lockdown measures. The survey was administered during periods of lockdown that were associated with significant restrictions to the residents of each nation (ie, April 21 to May 18, 2020 in the USA; April 30 to May 31, 2020 in the UK; April 21 to May 10, 2020 in France; May 1 to June 20, 2020 in Australia).

### Data analysis

Data were screened for univariate outliers using standardized scores (*z* > ± 3.29) and multivariate outliers using the Mahalanobis distance test (*p* <  0.001) [34]. The parametric assumptions that underlie mixed-model (M)ANOVA were examined [34]. PA (planned and unplanned), average steps per day and sedentary behavior (sitting and screen time) were analyzed by use of 2 (lockdown [pre vs. during]) × 4 (nation) × 4 (age group [18–29 years vs. 30–44 years vs. 45–59 years vs. ≥ 60 years]) (M)ANOVAs to address *H*_1_ and *H*_2_. Mental health was analyzed using a 2 (lockdown) × 4 (nation) × 2 (sex) ANOVA to address *H*_3_. Step-down *F* tests were Bonferroni adjusted, as were pairwise or multiple comparisons when used to identify where differences lay.

## Results

Details of data screening and diagnostics can be found in Additional file 2.

### Planned and unplanned PA

**Fig. 1:**
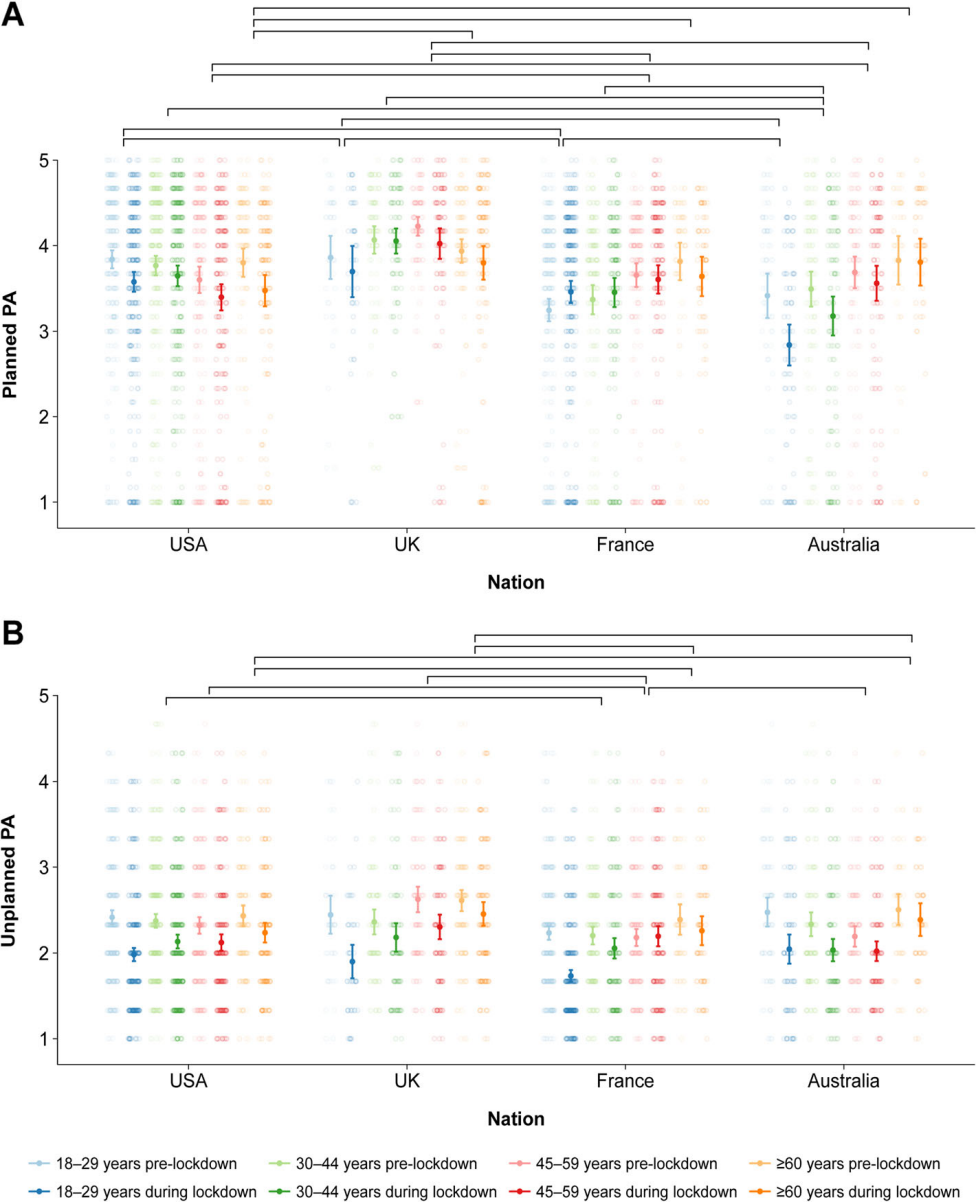
Stacked dotplot representing the higher-order interaction of lockdown × nation × age group for Brunel Lifestyle Physical Activity Questionnaire subscales. *M*, 95% CIs and density distributions are displayed for planned (**a**) and unplanned (**b**) dimensions of physical activity, pre- and during lockdown for each nation and age group. The values plotted are predicated on estimated marginal means. Brackets denote significant differences within the higher-order interaction. *p* < 0.001

A higher-order interaction of lockdown × nation × age group emerged with step-down *F* tests indicating that the interaction reached significance both for planned and unplanned PA (Table 1; Fig. 1a, b). There were significant two-way interactions of lockdown × nation and nation × age group for planned PA, with the USA and Australia showing a decline from pre- to during lockdown, whereas the UK and France did not. Australian young adults reported the lowest scores for planned PA across nations, whereas in the two middle-aged groups, the UK recorded the highest scores of all nations. Notably, French young adults were the only group to report an increase in planned PA from pre- to during lockdown.

**Table 1:** Inferential statistics for all dependent variables

Variable	Pillai’s Trace	***F***	***df***	***p***	η_p_^**2**^
Planned and unplanned PA					
Lockdown × nation × age group	0.02	2.80	18,5028	< 0.001	.01
Lockdown × nation	0.01	4.36	6,5028	< 0.001	.01
Lockdown × age group	0.02	9.34	6,5028	< 0.001	.01
Nation × age group	0.03	3.91	18,5028	< 0.001	.01
Lockdown	0.08	113.83	2,2513	< 0.001	.08
Nation	0.03	11.41	6,5028	< 0.001	.01
Age group	0.02	7.62	6,5028	< 0.001	.01
Average steps per day					
Lockdown × nation × age group	NA	1.88	9,1059	0.051	.02
Lockdown × nation	NA	3.88	3,1059	0.009	.01
Lockdown × age group	NA	23.48	3,1059	< 0.001	.06
Nation × age group	NA	1.41	9,1059	0.181	.01
Lockdown	NA	182.09	1,1059	< 0.001	.15
Nation	NA	13.50	3,1059	< 0.001	.04
Age group	NA	4.62	3,1059	0.003	.01
Sedentary behavior					
Lockdown × nation × age group	0.01	0.90	18,4974	0.586	.00
Lockdown × nation	0.02	7.18	6,4974	< 0.001	.01
Lockdown × age group	0.03	13.47	6,4974	< 0.001	.02
Nation × age group	0.02	2.43	18,4974	0.001	.01
Lockdown	0.23	375.72	2,2486	< 0.001	.23
Nation	0.01	3.43	6,4974	0.002	.00
Age group	0.05	20.94	6,4974	< 0.001	.03
Mental health					
Lockdown × nation × sex	NA	1.00	3,2524	0.393	.00
Lockdown × nation	NA	23.05	3,2524	< 0.001	.03
Lockdown × sex	NA	14.90	1,2524	< 0.001	.01
Nation × sex	NA	0.91	3,2524	0.433	.00
Lockdown	NA	294.60	1,2524	< 0.001	.11
Nation	NA	8.28	3,2524	< 0.001	.01
Sex	NA	14.56	1,2524	< 0.001	.01

*NA* not applicable, *PA* physical activity

For unplanned PA there were interactions of lockdown × age group and nation × age group. Although there was a decline in PA from pre- to during lockdown for all age groups, the decline was greatest among young adults, which led to the significant interaction. French young adults exhibited the lowest levels of unplanned PA across nations, whereas the UK had the highest recorded scores for upper middle-aged adults. Among older adults, unplanned PA scores were higher in the UK than in the USA and France.

Omnibus statistics for lockdown indicated a significant reduction in the composite PA variable from pre- to during lockdown (Table 1). A main effect of nation emerged for planned PA (Table 1) and pairwise comparisons indicated that all nations differed with the exception of France vs. Australia and France vs. USA. A main effect of nation was also observed for unplanned PA (Table 1) with pairwise comparisons showing differences between each pair of nations, except pairings with Australia. A main effect of age group was observed for both planned and unplanned PA (Table 1), with differences in planned PA for young adults when compared to upper middle-aged (*p* = 0.001) and older adults (*p* = 0.001). For unplanned PA, older adults differed from all other age groups.

### Average steps per day

**Fig. 2:**
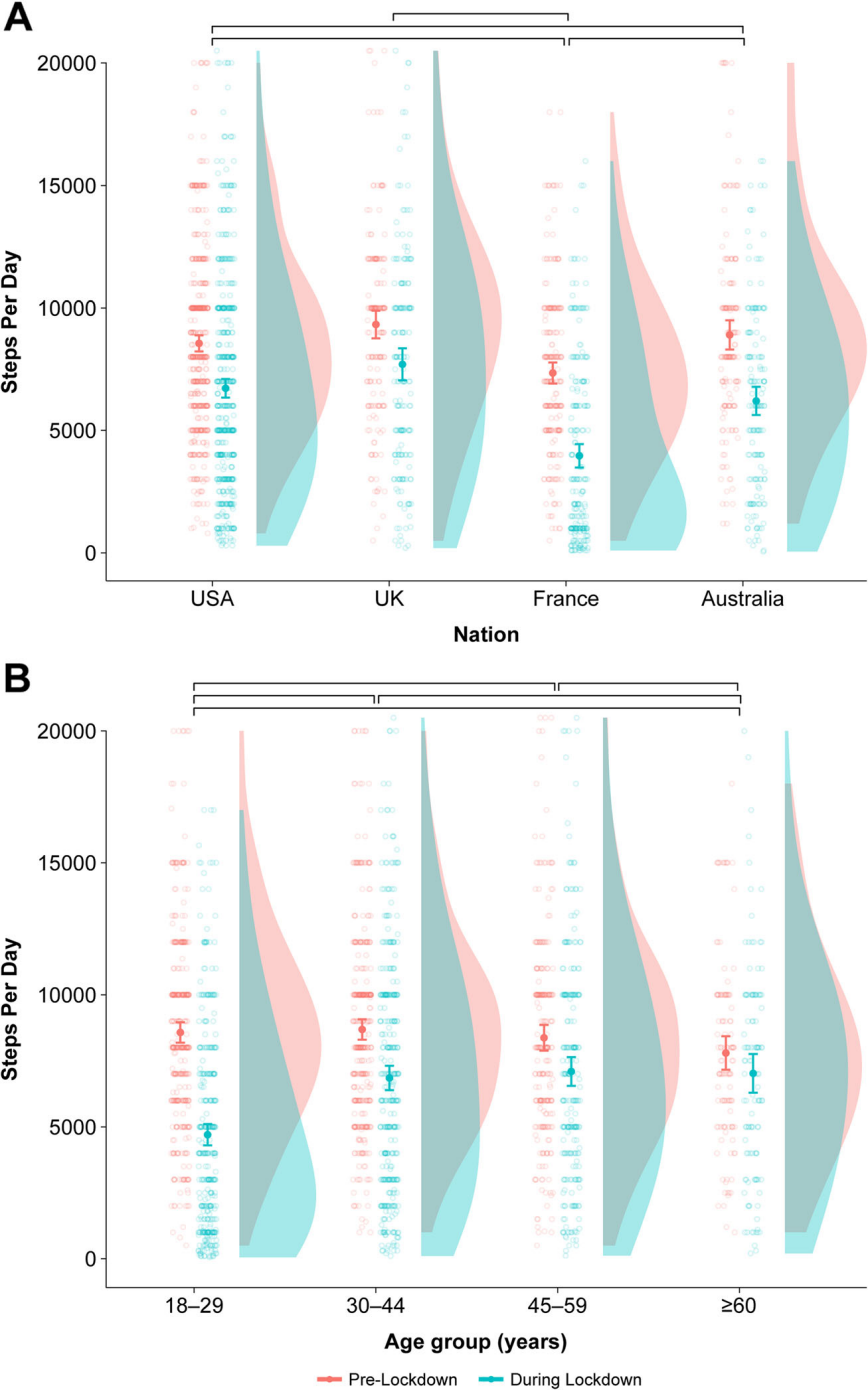
Raincloud plot representing two-way interactions of lockdown × nation (**a**) and lockdown × age group (**b**) for steps per day. *M*, 95% CIs and density distributions are displayed pre- and during lockdown for each nation (**a**) and age group (**b**). The values plotted are predicated on estimated marginal means. Brackets denote significant differences for each two-way interaction. *p* = 0.009 (**a**), *p* < 0.001 (**b**)

ANOVA for steps per day indicated that the higher-order interaction was marginally non-significant, although both two-way interactions (lockdown × nation and lockdown × age group) reached significance (Table 1; Fig. 2a, b). Notably, in all nations, there was a significant reduction in steps per day reported from pre- to during lockdown (ie, a main effect of lockdown). The lockdown × age group interaction indicated no change from pre- to during lockdown for older adults, but a significant decline for all other (younger) age groups, with the most marked reduction evident in young adults (Fig. 2b). The two-way nation × age group interaction was non-significant (Table 1).

There was a main effect of nation, with multiple comparisons showing that the UK daily step count was higher than in the USA (*p* = 0.015), France (*p* <  0.001) and Australia (*p* = 0.044); and higher in the USA and Australia than France (*p* <  0.001). There was a main effect of age group with multiple comparisons indicating differences between young and lower-middle aged adults (*p* <  0.001), and young and upper-middle aged adults (*p* <  0.001).

### Sedentary behavior

**Fig. 3:**
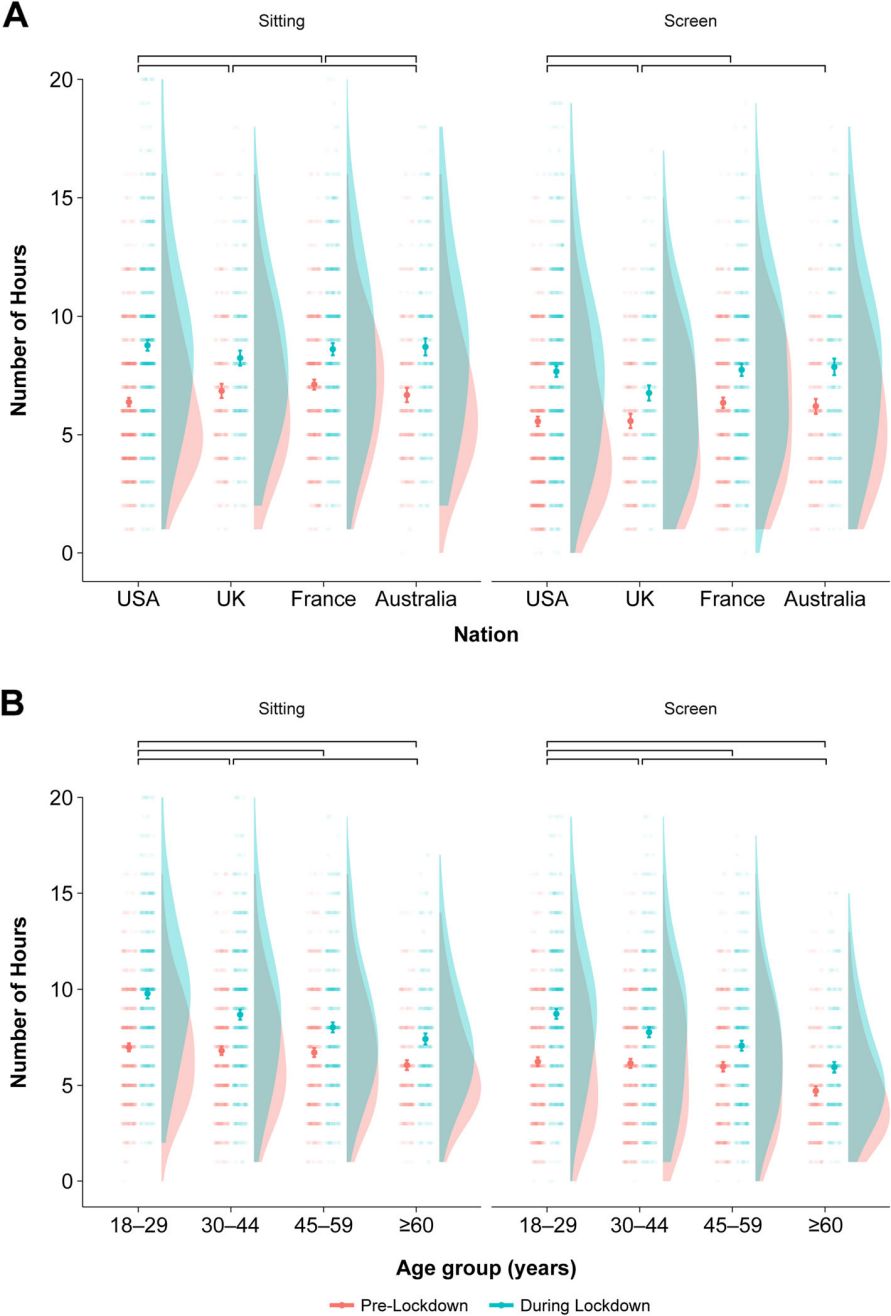
Raincloud plot representing two-way interactions of lockdown × nation (**a**) and lockdown × age group (**b**) for sedentary behavior. *M*, 95% CIs and density distributions are displayed for sitting and screen time, pre- and during lockdown for each nation (**a**) and age group (**b**). The values plotted are predicated on estimated marginal means. Brackets denote significant differences for each two-way interaction. *p*s < 0.001

The higher-order interaction of lockdown × nation × age group was non-significant. However, the omnibus two-way lockdown × nation interaction reached significance (Table 1). Step-down *F* tests indicated that the two-way interaction held for sitting and screen time (Fig. 3a). The interaction for sitting time was accounted for by a larger difference from pre- to during lockdown in the USA sample compared to France. The comparable interaction for screen time was accounted for by a larger difference from pre- to during lockdown in the USA compared to the UK and France. Moreover, there was a larger pre- to during lockdown difference in Australia than in the UK (Fig. 3a).

There was a significant omnibus two-way interaction of lockdown × age group for both sitting and screen time (Table 1). The *F* test for sitting time showed that the interaction could be attributed to a greater difference among young adults from pre- to during lockdown, compared to the other age groups. Moreover, the lower middle-aged adults had a greater difference from pre- to during lockdown than older adults (Fig. 3b). The *F* test for screen time showed that the interaction was driven by a greater difference among young adults from pre- to during lockdown compared to other age groups (Fig. 3b).

Omnibus statistics indicated a significant main effect of lockdown, associated with a large effect size (Table 1). Increases in sitting and screen time were confirmed in follow-up *F* tests. There was also a main effect of age group, associated with a small effect size (Table 1). Pairwise comparisons showed that young adults reported more sitting time than the other three age groups. Moreover, both the lower and upper middle-aged groups reported greater sitting time than older adults (*p*s <  0.001).

### Mental health

**Fig. 4:**
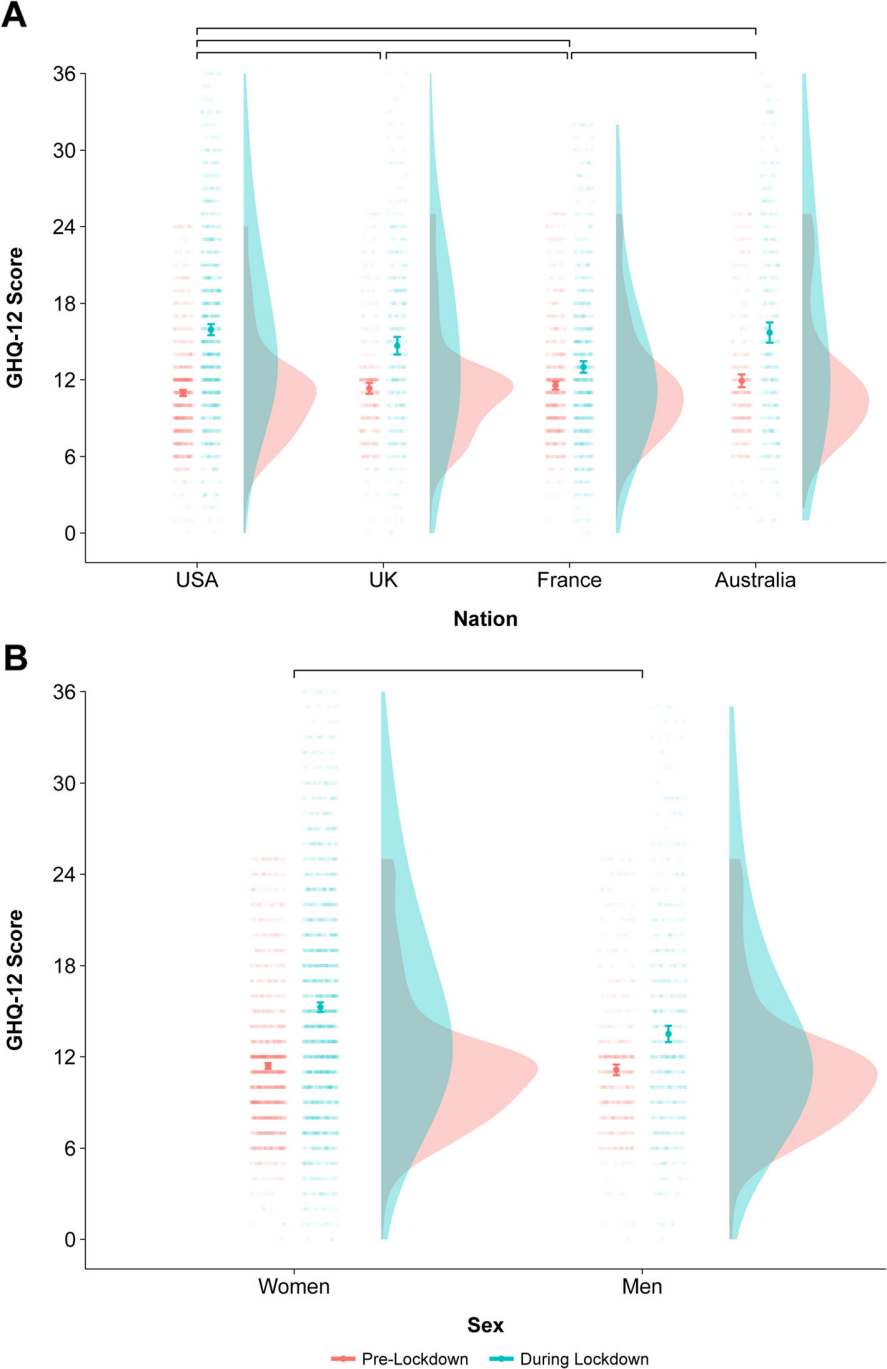
Raincloud plot representing two-way interactions of lockdown × nation (**a**) and lockdown × sex (**b**) for General Health Questionnaire-12 scores. *M*, 95% CIs and density distributions are displayed pre- and during lockdown for each nation (**a**) and sex (**b**). The values plotted are predicated on estimated marginal means. Brackets denote significant differences for each two-way interaction. *p*s < 0.001

ANOVA for GHQ-12 scores indicated no higher-order interaction of lockdown × nation × sex (Table 1). There were, however, significant two-way interactions of lockdown × nation and lockdown × sex. The lockdown × nation interaction can be attributed to the emergence of large differences in GHQ-12 scores from pre- to during lockdown in the USA, UK, and Australia, but only a small difference in France (Fig. 4a). The lockdown × sex interaction showed that lockdown was associated with a greater decrement in the mental health of women compared to men (Fig. 4b).

Main effects emerged for lockdown (pre < during) and nation (Table 1), with pairwise comparisons indicating that the USA and Australia samples reported higher scores (ie, worse mental health) than France (*p* = 0.001 and *p* <  0.001, respectively). Finally, there was a main effect of sex with pairwise comparisons indicating that women reported higher GHQ-12 scores than men (*p* <  0.001; *M*_diff_ = 0.91).

## Discussion

The primary aim of the present study was to investigate effects of COVID-19 lockdown across four Western nations with specific reference to PA, sedentary behavior, and mental health. The planned and unplanned dimensions of PA, as well as recorded steps per day, showed a significant reduction from pre- to during lockdown (Figs. 1b and 2b). A lockdown × age group interaction emerged for unplanned PA, which can be attributed to a steep decline among young adults, and so *H*_1_ is only partially accepted. It is notable that across the four nations, daily step count decreased by ~ 2000 steps.

An increase in sedentary behavior (ie, sitting and screen time) was observed from pre- to during lockdown. Lockdown × age group interactions emerged for both variables, which can be attributed to marked increases among young adults and older adults (Fig. 3b), and so *H*_2_ is not accepted. A decrement in mental health was observed from pre- to during lockdown, represented by increases in GHQ-12 scores. Women exhibited a more pronounced decline in their mental health during lockdown when compared to men (ie, a lockdown × sex interaction emerged), providing support for *H*_3_.

The present data indicate clearly that the phenomenon of lockdown in Western nations had negative consequences for planned and unplanned PA, as well as steps per day. Starting with planned PA, it is worth stressing that many exercise facilities were forced to close during lockdown. Consequently, some Western governments, such as those of the UK and Australia strongly encouraged regular daily exercise [35, 36]. Moreover, technology-mediated exercise programs gained huge popularity during the course of the pandemic [37]. A decline in planned PA emerged in the USA, UK, and Australia; this being least pronounced in the UK where strong encouragement was given for daily, socially distanced exercise [35].

It is notable that no difference emerged from pre- to during lockdown in the France sample. However, the French started with a much lower base of planned PA, which remained stable when lockdown was imposed (Fig. 1a). The implications for the French are that their nation’s base levels of PA, particularly among young and lower middle-aged adults, are so low that there is a case for far stronger public messaging to promote PA. Nonetheless, France has among the lowest levels of obesity among European nations [38], hinting that the population espouses relatively healthy eating habits [39]. To further investigate this notion, we conducted an a posteriori analysis into BMI scores across nations, which showed that the France sample exhibited the lowest scores (*F*_3, 2520_ = 58.70; ƞ_p_^2^ = .07; France [*M* = 23.90, *SD* = 4.29] < UK [*M* = 25.46, *SD* = 4.96] < USA [*M* = 26.63, *SD* = 5.46] < Australia [*M* = 27.64, *SD* = 5.96]).

For unplanned PA, the expected decline during lockdown was moderated by nation and age-group membership in combination (ie, a three-way interaction; Fig. 1b). Generally, young adults engaged in less unplanned PA during lockdown than other age groups, with the most marked decline evident in Europe (see UK and France stacked dotplots in Fig. 1b). An interesting aspect of the unplanned PA findings was that no differences emerged from pre- to during lockdown in the France and Australia samples for older adults. In the UK sample, the smallest decline was evident in older adults. This trend suggests that, during lockdown, people of working age were far less able to take advantage of the health benefits associated with incidental activity, such as ascending a flight of stairs in an office building. Given the lack of opportunity for unplanned PA among people of working age during lockdown, they would be well advised to schedule additional PA (ie, planned PA) into their daily routine [40].

Steps per day data indicated a reduction in steps equating to a daily energy expenditure of ~ 100 kcal across the four nations; roughly equivalent to a weight gain of 1.50 kg over lockdown, which has been reported elsewhere [41]. Other recent pandemic-related studies from Italy and Spain have reported lockdown-related weight gains of ~ 2 kg [41, 42]. The significant lockdown × age group interaction (Table 1) indicated a sharp decline in the daily step count of young adults from pre- to during lockdown (*M*_diff_ = 4185.67). This is just under half the number of steps that this age group would be recommended to take for optimal physical health [43]. It is notable that France and Australia showed the steepest declines in steps per day (*M*_diff_ = 2586.26, *M*_diff_ = 2554.21, respectively). This is unsurprising given government messages in densely populated areas, such as Paris, requiring people not to leave their homes except for “essential purposes”. Twenty-three participants from the France sample were from the Paris area and another 215 were from large metropolitan areas.

As expected, when people are forced to stay inside their homes, lockdown resulted in a ~ 2-h increase in reported sitting time (Fig. 3a). This finding is consistent with other studies conducted in Western nations [44, 45], albeit some studies report as much as a ~ 3-h increase [46]. The significant lockdown × age group interaction showed that the increase in sitting time was greatest among young and lower middle-aged adults. This suggests that these groups might be more active in non-pandemic times (eg, through walking to work, moving around their workplace, and dancing at social events).

The lockdown × nation interaction showed the increase in sitting time in the USA and Australia to be more pronounced than that in the UK and France. Notably, there is much less of a culture of walking or cycling as a mode of transport in the USA and Australia—vast countries in which per capita car ownership is high [47]—when compared to European countries such as the UK and France, which have a long-established culture of active travel [48]. Interestingly, the statistical trends found in screen time mirror those found in sitting time (Fig. 3b). Accordingly, much of the time spent sitting entailed the use of screen-based technology.

Findings for sedentary behavior are among the most compelling when juxtaposed against the backdrop of dangerously high levels of sedentary behavior in the pre-pandemic era [5]. The implications for cardiometabolic health are manifold and hint at the importance of encouraging the public to engage in regular bouts of PA during periods of lockdown. Failure to do so will increase the number of lives claimed by COVID-19 with many additional lives lost to obesity, type 2 diabetes, and heart disease [4]. The most worrisome findings come in examining jointly the PA and sedentary behavior data for young adults, as this group appears to have been most adversely affected by lockdown [10, 18].

The combined findings for PA and sedentary behavior paint a picture of large swaths of young adults in Western countries who give insufficient attention to their PA needs. It is worth highlighting that our findings mirror those of other recent multination studies [22, 49]. The pandemic has served to shine a light on underlying attitudes toward PA that will need to be addressed in the post-pandemic era. It has become clear from a welter of epidemiological studies that regular PA can have a prophylactic effect in the face of COVID-19 (ie, in terms of the most severe symptoms), as well as many other infections, such as influenza and pneumonia [50, 51]. Thus, siting health and PA as a centerpiece of school curricula—with appropriate theoretical and practical content—is a societal imperative [52].

The GHQ-12 provides insight into common mental disorders (CMDs) such as anxiety, depression, and social dysfunction. Although usually less disabling than major psychiatric disorders, CMDs are more prevalent (eg, one in six adults in England) [53], and are thus likely to have greater societal impact. We predicted that conditions of lockdown would elicit declines in mental health that would be greater among women, which is precisely what is shown in the lockdown × sex interaction (Fig. 4b), and replicated in many similar studies [eg, [18, 54]]. The findings are also notable for variations in mental health across Western nations. Pre-lockdown, a small difference was evident between the USA and Australia, with poorer mental health scores in Australia (Fig. 4a). During lockdown, the USA sample reported the largest decline in mental health scores, with differences emerging between the USA and both the UK and France.

Notably, the French data show the greatest stability in mental health from pre- to during lockdown (Fig. 4a). This might be attributed, in part, to 40.6% of French respondents residing in rural locations. Although lockdowns are immediately apparent and perhaps anxiety provoking in urban environments, there are fewer noticeable changes in rural environments, where population density is much lower. The decrease in mental health in the USA sample is of particular concern (*M*_diff_ = 4.43); perhaps the uncertainties associated with an impending presidential election coupled with the lack of an economic safety net for large segments of the population, contributed to this finding [14]. It is vital that further work is conducted into the association between population density and mental health; times of crisis such as war, famine, and now a pandemic, bring the mental well-being of urban populations into sharp focus [55].

The decline in women’s mental health during lockdown, regardless of the nation in which they resided, is also worrisome (Fig. 4b). There was little that state governments could do to mitigate against the competing demands of full-time work, home schooling, and domestic responsibilities that many women faced [56]. It should be added that the demands of looking after children or elderly relatives may have prevented many women from engaging in exercise activities, which are known to contribute positively to mental health [9]. A clear implication is that state governments should consider women-friendly policies pertaining to flexible working, childcare, and mental health helplines in any future pandemic [18, 57].

The present study employed an online survey method to collect data across four Western nations. Although the way in which the data were collected was standardized and entailed adjustments to render each survey culturally specific, it should be noted that a self-selection bias does pervade scientific work of this nature. In examining our demographic data (Additional file 1: Table S1), it is clear that (a) relatively few men chose to complete the survey (23.5%), (b) it attracted relatively few responses from those living in rural areas (34.2%), with the notable exception of French respondents (40.6%), and (c) it largely failed to reach individuals representing lower socio-economic groups (4.7%). The self-selection bias should thus be borne in mind when attempting to generalize the present findings to the populations that were sampled, as well as to the populations of other Western nations. A further limitation pertains to the retrospective recall of planned/unplanned PA, sedentary behavior, and mental health in relation to the period prior to lockdown. Future online studies of this nature might use incentives for the hard-to-reach contingents of the population, as well as adopt a longitudinal approach to circumvent the need for retrospective recall.

Participant incentives and a longitudinal approach were not possible in the present study, as the research team responded nimbly to the initial spate of national lockdowns and the circumstances for a ‘natural experiment’ [26]. In the limited time window open to the research team, we aimed to collect as much data as we could, rather than be guided by an a priori power analysis. Given the wealth of data now published [10, 11, 13], investigators of future lockdowns will have effect sizes from several nations to inform their estimations of sample size. We put ethical approval applications through our respective institutions immediately after national lockdowns were declared, without having time to apply for funding that would have provided incentives for participants, and without knowing that there would be multiple lockdowns in the months ahead. At the time that our study was initiated, the general consensus was that lockdowns would be a relatively short-lived phenomenon [58]. A further limitation concerns the low alpha estimates for the three-item unplanned PA scale (0.52 pre-lockdown and 0.64 during lockdown).

## Conclusions

We took an international perspective in the assessment of how national lockdowns influenced PA, sedentary behavior, and mental health. The most striking finding is that lockdown led to detriments in PA and mental health, while sharp increases in sedentary behavior were also recorded. Across nations, it was reported that ~ 2000 fewer daily steps were taken, which equates to the non-expenditure of ~ 100 kcal. Changes in PA and sedentary behavior among young adults are of particular concern, as is the reduction in the mental health of Americans across all age groups. It is important for policy makers to address the deleterious effect of lockdowns on women’s mental health, perhaps through adopting women-friendly policies for any future lockdown.

## Supplementary Information

**Additional file 1: Table S1.** Anthropomorphic, demographic and health characteristics of the present sample.

**Additional file 2.** Data screening and diagnostics.

## Data Availability

All data relevant to the study can be obtained from this link: https://figshare.com/s/4b070f0e52af69ef94ef.

## Abbreviations

BLPAQ: Brunel Lifestyle Physical Activity Questionnaire
CMDs: Common mental disorders
GHQ-12: General Health Questionnaire-12
PA: Physical activity
