# Supplementary material for: Physical activity and mental well-being under COVID-19 lockdown: a cross-sectional multination study
Source: BMC Public Health. 2021 May 27;21:988. doi: 10.1186/s12889-021-10931-5 (PMC8154111; doi:10.1186/s12889-021-10931-5)
Supplement: Supplementary file 1 — Additional file 1: Table S1. Anthropomorphic, demographic and health characteristics of the present sample. [file 12889_2021_10931_MOESM1_ESM.docx]

Physical Activity and Mental Well-Being Under COVID-19 Lockdown:
A Cross-Sectional Multination Study

Costas I. Karageorghis^1^, Jonathan M. Bird^2^, Jasmin C. Hutchinson^3^, Mark Hamer^4^, Yvonne N. Delevoye-Turrell^5^, Ségolène M. R. Guérin^5^, Elizabeth M. Mullin^3^, Kathleen T. Mellano^3^, Renée L. Parsons-Smith^6,7^, Victoria R. Terry^8^, Peter C. Terry^9^

^1^Department of Life Sciences, Brunel University London, United Kingdom

^2^Department of Science, Innovation, Technology, and Entrepreneurship,
University of Exeter, United Kingdom

^3^Department of Exercise Science and Athletic Training,
Springfield College, United States of America

^4^Institute of Sport, Exercise & Health, Research Department of Targeted Intervention, University College London, United Kingdom

^5^Department of Cognitive Sciences and Affective Sciences, University of Lille, France

^6^School of Psychology and Counselling, University of Southern Queensland, Australia

^7^School of Social Sciences, University of the Sunshine Coast, Australia

^8^School of Nursing and Midwifery, University of Southern Queensland, Australia

^9^Division of Research and Innovation, University of Southern Queensland, Australia

Correspondence concerning this article should be addressed to Costas I. Karageorghis, Department of Life Sciences, Brunel University London, United Kingdom, UB8 3PH.
Email: costas.karageorghis@brunel.ac.uk, Tel: +44 (0)1895 266476.

**Additional File 1**

**Table S1** Anthropomorphic, demographic and health characteristics of the present sample

|  | **Total Sample**  **(*N* = 2,541; 100%)** | | **USA**  **(*n* = 1,029; 40.5%)** | | **UK**  **(*n* = 392; 15.4%)** | | **France**  **(*n* = 734; 28.9%)** | | **Australia**  **(*n* = 386; 15.2%)** | |
| --- | --- | --- | --- | --- | --- | --- | --- | --- | --- | --- |
| **Variable** | ***M*** | ***SD*** | ***M*** | ***SD*** | ***M*** | ***SD*** | ***M*** | ***SD*** | ***M*** | ***SD*** |
| Height (m) |  |  |  |  |  |  |  |  |  |  |
| Women | 1.65 | 0.07 | 1.65 | 0.07 | 1.64 | 0.07 | 1.66 | 0.06 | 1.65 | 0.08 |
| Men | 1.79 | 0.07 | 1.79 | 0.07 | 1.78 | 0.07 | 1.79 | 0.06 | 1.79 | 0.09 |
| Total | 1.68 | 0.09 | 1.68 | 0.09 | 1.67 | 0.09 | 1.69 | 0.08 | 1.69 | 0.10 |
| Weight (kg) |  |  |  |  |  |  |  |  |  |  |
| Women | 70.11 | 16.13 | 72.49 | 16.60 | 68.57 | 13.42 | 64.93 | 13.48 | 75.54 | 19.14 |
| Men | 83.82 | 14.92 | 86.00 | 14.77 | 80.18 | 12.48 | 79.14 | 13.04 | 89.52 | 16.91 |
| Total | 73.34 | 16.92 | 75.60 | 17.19 | 70.86 | 14.00 | 68.34 | 14.68 | 79.43 | 19.66 |
| Body mass index (BMI) |  |  |  |  |  |  |  |  |  |  |
| Women | 25.68 | 5.57 | 26.59 | 5.72 | 25.49 | 5.13 | 23.63 | 4.48 | 27.45 | 6.30 |
| Men | 26.20 | 4.41 | 26.77 | 4.48 | 25.31 | 4.27 | 24.74 | 3.48 | 28.03 | 4.84 |
| Total | 25.81 | 5.33 | 26.63 | 5.46 | 25.46 | 4.96 | 23.90 | 4.29 | 27.64 | 5.96 |
|  | ***n*** | **%** | ***n*** | **%** | ***n*** | **%** | ***n*** | **%** | ***n*** | **%** |
| Sex |  |  |  |  |  |  |  |  |  |  |
| Women |  |  |  |  |  |  |  |  |  |  |
| 18–29 years | 589 | 77.9 | 239 | 75.6 | 33 | 70.2 | 243 | 79.2 | 74 | 86.0 |
| 30–44 years | 565 | 77.7 | 266 | 81.1 | 74 | 74.0 | 134 | 77.5 | 91 | 72.2 |
| 45–59 years | 486 | 75.6 | 176 | 77.2 | 93 | 83.0 | 132 | 71.7 | 85 | 71.4 |
| ≥ 60 years | 298 | 73.4 | 105 | 69.5 | 114 | 86.4 | 49 | 70.0 | 30 | 56.6 |
| Total | 1,938 | 76.5 | 786 | 76.8 | 314 | 80.3 | 558 | 76.0 | 280 | 72.9 |
| Men |  |  |  |  |  |  |  |  |  |  |
| 18–29 years | 167 | 22.1 | 77 | 24.4 | 14 | 29.8 | 64 | 20.8 | 12 | 14.0 |
| 30–44 years | 162 | 22.3 | 62 | 18.9 | 26 | 26.0 | 39 | 22.5 | 35 | 27.8 |
| 45–59 years | 157 | 24.4 | 52 | 22.8 | 19 | 17.0 | 52 | 28.3 | 34 | 28.6 |
| ≥ 60 years | 108 | 26.6 | 46 | 30.5 | 18 | 13.6 | 21 | 30.0 | 23 | 43.4 |
| Total | 594 | 23.5 | 237 | 23.2 | 77 | 19.7 | 176 | 24.0 | 104 | 27.1 |
| Setting |  |  |  |  |  |  |  |  |  |  |
| Rural |  |  |  |  |  |  |  |  |  |  |
| 18–29 years | 241 | 31.9 | 61 | 19.2 | 13 | 28.3 | 109 | 35.5 | 58 | 67.4 |
| 30–44 years | 206 | 28.2 | 36 | 10.9 | 24 | 24.0 | 60 | 34.7 | 86 | 68.3 |
| 45–59 years | 267 | 41.3 | 43 | 18.7 | 43 | 38.4 | 96 | 52.2 | 85 | 70.8 |
| ≥ 60 years | 154 | 37.7 | 33 | 21.9 | 52 | 39.1 | 33 | 47.1 | 36 | 66.7 |
| Total | 868 | 34.2 | 173 | 16.8 | 132 | 33.8 | 298 | 40.6 | 265 | 68.7 |
| Urban |  |  |  |  |  |  |  |  |  |  |
| 18–29 years | 515 | 68.1 | 256 | 80.8 | 33 | 71.7 | 198 | 64.5 | 28 | 32.6 |
| 30–44 years | 524 | 71.8 | 295 | 89.1 | 76 | 76.0 | 113 | 65.3 | 40 | 31.7 |
| 45–59 years | 379 | 58.7 | 187 | 81.3 | 69 | 61.6 | 88 | 47.8 | 35 | 29.2 |
| ≥ 60 years | 254 | 62.3 | 118 | 78.1 | 81 | 60.9 | 37 | 52.9 | 18 | 33.3 |
| Total | 1,672 | 65.8 | 856 | 83.2 | 259 | 66.2 | 436 | 59.4 | 121 | 31.3 |
| Continued | | | | | | | | | | |
|  | | | | | | | | | | |

**Table 1** Continued

|  | **Total Sample**  **(*N* = 2,541; 100%)** | | **USA**  **(*n* = 1,029; 40.5%)** | | **UK**  **(*n* = 392; 15.4%)** | | **France**  **(*n* = 734; 28.9%)** | | **Australia**  **(*n* = 386; 15.2%)** | |
| --- | --- | --- | --- | --- | --- | --- | --- | --- | --- | --- |
| **Variable** | ***n*** | **%** | ***n*** | **%** | ***n*** | **%** | ***n*** | **%** | ***n*** | **%** |
| Socio-economic status |  |  |  |  |  |  |  |  |  |  |
| Upper |  |  |  |  |  |  |  |  |  |  |
| 18–29 years | 391 | 69.3 | 206 | 74.1 | 26 | 63.4 | 134 | 69.8 | 25 | 47.2 |
| 30–44 years | 533 | 78.3 | 291 | 92.1 | 74 | 76.3 | 113 | 67.3 | 55 | 55.0 |
| 45–59 years | 454 | 75.5 | 186 | 86.5 | 73 | 69.5 | 119 | 66.5 | 76 | 74.5 |
| ≥ 60 years | 297 | 76.9 | 118 | 86.8 | 90 | 69.8 | 52 | 75.4 | 37 | 71.2 |
| Total | 1,675 | 75.0 | 801 | 84.8 | 263 | 70.7 | 418 | 68.8 | 193 | 62.9 |
| Middle |  |  |  |  |  |  |  |  |  |  |
| 18–29 years | 138 | 24.5 | 64 | 23.0 | 9 | 22.0 | 55 | 28.6 | 10 | 18.9 |
| 30–44 years | 117 | 17.2 | 22 | 7.0 | 21 | 21.6 | 54 | 32.1 | 20 | 20.0 |
| 45–59 years | 120 | 20.0 | 22 | 10.2 | 27 | 25.7 | 58 | 32.4 | 13 | 12.7 |
| ≥ 60 years | 77 | 19.9 | 13 | 9.6 | 37 | 28.7 | 16 | 23.2 | 11 | 21.2 |
| Total | 452 | 20.3 | 121 | 12.8 | 94 | 25.3 | 183 | 30.1 | 54 | 17.6 |
| Lower |  |  |  |  |  |  |  |  |  |  |
| 18–29 years | 35 | 6.2 | 8 | 2.9 | 6 | 14.6 | 3 | 1.6 | 18 | 34.0 |
| 30–44 years | 31 | 4.6 | 3 | 0.9 | 2 | 2.1 | 1 | 0.6 | 25 | 25.0 |
| 45–59 years | 27 | 4.5 | 7 | 3.3 | 5 | 4.8 | 2 | 1.1 | 13 | 12.7 |
| ≥ 60 years | 12 | 3.1 | 5 | 3.7 | 2 | 1.6 | 1 | 1.4 | 4 | 7.7 |
| Total | 105 | 4.7 | 23 | 2.4 | 15 | 4.0 | 7 | 1.2 | 60 | 19.5 |
| Health conditions present |  |  |  |  |  |  |  |  |  |  |
| Yes | 607 | 24.1 | 296 | 29.0 | 105 | 27.0 | 110 | 15.2 | 96 | 25.3 |
| No | 1,909 | 75.9 | 726 | 71.0 | 284 | 73.0 | 616 | 84.8 | 283 | 74.7 |
| Disabilities |  |  |  |  |  |  |  |  |  |  |
| Yes | 113 | 4.5 | 50 | 4.9 | 35 | 9.0 | 20 | 2.7 | 8 | 2.2 |
| No | 2,399 | 95.5 | 971 | 95.1 | 354 | 91.0 | 712 | 97.3 | 362 | 97.8 |
| COVID-19 symptoms |  |  |  |  |  |  |  |  |  |  |
| Yes | 220 | 8.7 | 80 | 7.8 | 41 | 10.5 | 73 | 10.0 | 26 | 6.7 |
| No | 2,314 | 91.3 | 945 | 92.2 | 350 | 89.5 | 659 | 90.0 | 360 | 93.3 |
| COVID-19 diagnosis |  |  |  |  |  |  |  |  |  |  |
| Yes | 23 | 0.9 | 8 | 0.8 | 2 | 0.5 | 13 | 1.8 | NA | NA |
| No | 2,509 | 98.9 | 1,015 | 98.8 | 388 | 99.2 | 720 | 98.2 | 386 | 100.0 |
| Awaiting test results | 5 | 0.2 | 4 | 0.4 | 1 | 0.3 | NA | NA | NA | NA |
| Recovery from COVID-19 symptoms |  |  |  |  |  |  |  |  |  |  |
| Yes | 199 | 7.9 | 74 | 7.2 | 35 | 9.0 | 66 | 9.1 | 24 | 6.3 |
| No | 240 | 9.5 | 116 | 11.3 | 43 | 11.0 | 27 | 3.7 | 54 | 14.1 |
| Not relevant to me | 2,089 | 82.6 | 836 | 81.5 | 312 | 80.0 | 635 | 87.2 | 306 | 79.7 |
| Adherence to state government COVID-19 guidelines |  |  |  |  |  |  |  |  |  |  |
| Extremely unlikely | 97 | 3.8 | 43 | 4.2 | 14 | 3.6 | 22 | 3.0 | 18 | 4.7 |
| Somewhat unlikely | 57 | 2.2 | 25 | 2.4 | 2 | 0.5 | 15 | 2.0 | 15 | 3.9 |
| Neither likely nor unlikely | 38 | 1.5 | 9 | 0.9 | 1 | 0.3 | 24 | 3.3 | 4 | 1.0 |
| Somewhat likely | 548 | 21.6 | 186 | 18.1 | 40 | 10.2 | 243 | 33.1 | 79 | 20.5 |
| Extremely likely | 1,801 | 70.9 | 766 | 74.4 | 335 | 85.5 | 430 | 58.6 | 270 | 69.9 |

*NA* not applicable. In the interests of brevity, participants who responded with “prefer not to say” to any of the items listed in this table have been excluded. The socio-economic data use occupation as a proxy for socio-economic status and the classifications are derived through consolidation of occupation-related data from each nation. Race and ethnicity are not reported, as there were vastly different ways in which each nation’s government recorded such data. In France, we were not permitted to collate such data.
