# Supplementary material for: Physical activity and mental well-being under COVID-19 lockdown: a cross-sectional multination study
Source: BMC Public Health. 2021 May 27;21:988. doi: 10.1186/s12889-021-10931-5 (PMC8154111; doi:10.1186/s12889-021-10931-5)
Supplement: Supplementary file 2 — Additional file 2. Data screening and diagnostics. [file 12889_2021_10931_MOESM2_ESM.docx]

Physical Activity and Mental Well-Being Under COVID-19 Lockdown:
A Cross-Sectional Multination Study

Costas I. Karageorghis^1^, Jonathan M. Bird^2^, Jasmin C. Hutchinson^3^, Mark Hamer^4^, Yvonne N. Delevoye-Turrell^5^, Ségolène M. R. Guérin^5^, Elizabeth M. Mullin^3^, Kathleen T. Mellano^3^, Renée L. Parsons-Smith^6,7^, Victoria R. Terry^8^, Peter C. Terry^9^

^1^Department of Life Sciences, Brunel University London, United Kingdom

^2^Department of Science, Innovation, Technology, and Entrepreneurship,
University of Exeter, United Kingdom

^3^Department of Exercise Science and Athletic Training,
Springfield College, United States of America

^4^Institute of Sport, Exercise & Health, Research Department of Targeted Intervention, University College London, United Kingdom

^5^Department of Cognitive Sciences and Affective Sciences, University of Lille, France

^6^School of Psychology and Counselling, University of Southern Queensland, Australia

^7^School of Social Sciences, University of the Sunshine Coast, Australia

^8^School of Nursing and Midwifery, University of Southern Queensland, Australia

^9^Division of Research and Innovation, University of Southern Queensland, Australia

Correspondence concerning this article should be addressed to Costas I. Karageorghis, Department of Life Sciences, Brunel University London, United Kingdom, UB8 3PH.
Email: costas.karageorghis@brunel.ac.uk, Tel: +44 (0)1895 266476.

**Additional File 2**

**Data Screening and Diagnostics**

Outlier tests revealed 200 univariate outliers that were modified to be one unit larger or smaller than the next most extreme score in the distribution, until the corresponding *z*-scores fell within the range -3.29–3.29 [1]. There were 44 multivariate outliers identified in the BLPAQ and sedentary behaviour MANOVAs that were screened out of the corresponding analysis. We checked the distribution of each dependent variable within each cell of each analysis. Except for three cells that exhibited moderate negative skewness (1.7%) and 13 that exhibited moderate positive skewness (7.4%), normal distributions were evident in the remaining 160 cells (90.9%). Box’s M test was used to check for the equality of multiple variance–covariance matrices and this reached significance in all four analyses (*p*s < 0.001).

**References**

1. Tabachnick BG, Fidell LS. Using multivariate statistics. 7th ed. London, UK: Pearson Education; 2019.
